# Home Mechanical Ventilation in Childhood-Onset Hereditary Neuromuscular Diseases: 13 Years’ Experience at a Single Center in Korea

**DOI:** 10.1371/journal.pone.0122346

**Published:** 2015-03-30

**Authors:** Young Joo Han, June Dong Park, Bongjin Lee, Yu Hyeon Choi, Dong In Suh, Byung Chan Lim, Jong-Hee Chae

**Affiliations:** 1 Department of Pediatrics, Seoul National University College of Medicine, Seoul, Korea; 2 Pediatric Clinical Neuroscience Center, Seoul National University Children’s Hospital, Seoul, Korea; University of Bari, ITALY

## Abstract

**Introduction:**

Children with hereditary neuromuscular diseases (NMDs) are at a high risk of morbidity and mortality related to respiratory failure. The use of home mechanical ventilation (HMV) has saved the lives of many children with NMD but, due to a lack of studies, dependable guidelines are not available. We drew upon our experience to compare the various underlying NMDs and to evaluate HMV with regard to respiratory morbidity, the proper indications and timing for its use, and to develop a policy to improve the quality of home noninvasive ventilation (NIV).

**Methods:**

We retrospectively analyzed the medical records of 57 children with childhood-onset hereditary NMDs in whom HMV was initiated between January 2000 and May 2013 at Seoul National University Children's Hospital. The degree of respiratory morbidity was estimated by the frequency and duration of hospitalizations caused by respiratory distress.

**Results:**

The most common NMD was spinal muscular atrophy (SMA, n = 33). Emergent mechanical ventilation was initiated in 44% of the patients before the confirmed diagnosis, and the indicators of pre-HMV respiratory morbidity (e.g., extubation trials, hypoxia, hospitalizations, and intensive care unit stay) were greater in these patients than in others. The proportion of post-HMV hospitalizations (range, 0.00−0.52; median, 0.01) was lower than that of pre-HMV hospitalizations (0.02−1.00; 0.99) (*P* < 0.001). Eight patients were able to maintain home NIV. The main causes of NIV failure were air leakage and a large amount of airway secretions.

**Conclusions:**

The application of HMV helped reduce respiratory morbidity in children with childhood-onset hereditary NMD. Patients with SMA type I can benefit from an early diagnosis and the timely application of HMV. The choice between invasive and noninvasive HMV should be based on the patient’s age and NIV trial tolerance. Systematic follow-up guidelines provided by a multidisciplinary team are needed.

## Introduction

Children with childhood-onset hereditary neuromuscular diseases (NMDs) are at a high risk of morbidity and mortality related to neuromuscular respiratory failure. Respiratory muscle weakness resulting from NMD causes insufficient ventilation, nocturnal hypoventilation, and ineffective coughing. Bulbar dysfunction coexists in many cases. Consequently, patients with neuromuscular respiratory failure experience hypoxemia, hypercapnia, secretion retention, aspiration, atelectasis, and recurrent pneumonia. This condition threatens the quality of life as well its length [1].

Since its introduction in the 1980s, the use of home mechanical ventilation (HMV) has saved the lives of many children with NMD, even though it is a palliative therapy [2, 3]. By unburdening the respiratory muscles, HMV appears to enhance both quality of life and nutritional status. Proper respiratory support can also decelerate declines in pulmonary function [4–6].

Many pediatricians, however, are still hesitant to offer this treatment option to children with NMD for several reasons. Data regarding the underlying childhood-onset hereditary NMD, the conditions of the patients, and practical situations are still insufficient. Furthermore, dependable guidelines regarding the application of HMV to children with NMD are not available. In addition, the prognoses of the patients are not clear, especially if they require respiratory support in infancy or early childhood. Finally, many questions remain regarding ethical issues [7].

Although some reports have described HMV for children with chronic respiratory failure regardless of underlying pulmonary or neurological disorders [8–10], few have evaluated pediatric HMV for pure childhood-onset hereditary peripheral NMD. Thus far, the most commonly investigated NMD has been spinal muscular atrophy (SMA) [3]. Children with typical SMA type I are never able to sit up, and they die of respiratory failure before 2 years of age [11, 12]. SMA type I studies can provide information regarding HMV for infants or young children with NMD because children with SMA type I inevitably require respiratory support before 1 or 2 years of age [11]. HMV reportedly extends the life spans of children with SMA type I [13], and the quality of life estimations made by the care providers of children with SMA type I receiving HMV do not differ from those made by parents of other unaffected children in the same age group [14].

HMV was introduced in the late 1990s in South Korea, and the number of patients who depend on HMV is still increasing [15]. At our center, the demand for HMV for children with NMD has been increasing since 2000. The accumulation of pediatric NMD experience, the application of HMV to these conditions, the technological improvements in home ventilator systems, and governmental support for rare genetic diseases have contributed to this increase.

For the last 3 years, our center has attempted to apply home noninvasive ventilation (NIV) to young children with NMD more aggressively than in the past, adopting an up-to-date strategy of combined NIV plus mechanical in-exsufflator therapy [16]. NIV offers the attractive advantage of preventing discomfort, complications, and the potential risks of tracheostomy and, in addition, preserves the patient’s ability to verbalize. Mechanical in-exsufflator therapy is helpful because it addresses the secretion-related shortcomings of NIV [16, 17]. We do not assume, however, that NIV is always better than invasive ventilation for children with NMD because situations associated with acute respiratory failure during both invasive and noninvasive HMV are complicated. Clinicians need to take multiple host and environmental factors into account and select the best respiratory support option while remembering that young children are small and uncooperative. Therefore, we need to understand numerous factors associated with HMV for NMD and the past/present situation in our own center and country.

Here we present our 13-year experience with HMV for children with NMDs in South Korea. We focused on comparing the various underlying NMDs, the benefit of HMV with regard to respiratory morbidity, HMV timing and techniques, and long-term outcomes. Further, we reviewed both our successful and failed NIV experiences to determine the proper indications for its use and to improve the quality of home NIV. Finally, we provide practical guidelines for the application of HMV in children with NMDs.

## Patients and Methods

### Patient selection

We retrospectively analyzed the medical records of children who were diagnosed with childhood-onset hereditary NMD before 15 years of age at Seoul National University Children's Hospital and for whom HMV was initiated between January 2000 and May 2013. Our pediatric neurologists confirmed the diagnoses with pathological or genetic tests. Patients with Duchenne muscular dystrophy were excluded from the study because most of them at our center start to receive HMV after their adolescence.

Written informed consent of the participants was waived by the Institutional Review Board of Seoul National University College of Medicine and all the information from the clinical records of the patients was anonymized and de-identified prior to analysis. This study was reviewed and approved by the Institutional Review Board of Seoul National University College of Medicine (H-1210-042-433).

### Definitions

We defined HMV as mechanical ventilator support performed at home, invasive HMV as HMV through tracheostomy, noninvasive HMV or home NIV as HMV using full-face or nasal masks, and planned HMV as HMV that was initiated based on decisions made at outpatient clinics. The criteria for decision were sustained hypercapnia and recurrent admissions for management of hypoxia despite supplementary oxygen. We defined the time of clinical diagnosis of NMD as the first day that clinicians suspected NMD based on developmental history and neurological examination and the time of confirmed diagnosis as the time when the diagnosis was confirmed by genetic or pathologic studies.

We estimated the degree of respiratory morbidity by the frequency and duration of hospitalizations caused by respiratory distress, pre-HMV hospitalization as an admission related to respiratory distress that occurred from birth to the initiation of HMV, and post-HMV hospitalization as an admission related to respiratory problems that occurred after the initiation of HMV, such as a respiratory tract infection, tracheostomy-associated problems, and the need for augmentation of the HMV settings. We calculated the frequency of hospitalizations per year using the total number of pre- or post-HMV admissions divided by the age (years) at the start of HMV or the total duration (years) of HMV, respectively. We calculated the hospitalization proportion using the total cumulative days of pre- or post-HMV admissions divided by the age (days) at the start of HMV or the total duration (days) of HMV, respectively.

We defined an episode of hypoxia as any episode with oxygen saturation below 50% as measured by a pulse oximeter, desaturation plus bradycardia, cardiopulmonary arrest, or emergent tracheal intubation.

### Statistics

For statistical analysis, we used the Statistical Package for the Social Sciences software version 21.0 (IBM, USA; 2012). We used the Mann-Whitney test to compare median values, the paired sample *t*-test to compare pre- and post-HMV data from each patient, Fisher’s exact test to determine associations between 2 categorical variables, and Kaplan-Meier analysis to evaluate the cumulative rate of survival. We considered *P* < 0.05 as statistically significant.

## Results

### Patient characteristics

Table 1 lists the patients’ characteristics. There were 57 patients, and the most common NMD was SMA (n = 33, 58%). Table 2 shows the characteristics of the patients according to underlying NMD.

**Table 1:** Clinical Characteristics of Enrolled Patients.

Characteristics	No. (%) of patients (N = 57)
Male	31 (54)
Age at the start of HMV (months)	7.7 (2−158)
Time from confirmed diagnosis to the start of MV* (months)	0.6 (-13.4−113.3)
Diagnosis	
Spinal muscular atrophy	33 (58)
Type I	29 (51)
Type II	4 (7)
Congenital myopathy	14 (25)
Myotubular	5 (9)
Type I fiber atrophy	2 (4)
Fiber type disproportion	3 (5)
Nemaline rod	1 (2)
Unspecified	3 (5)
Congenital muscular dystrophy	8 (14)
Merosin-negative	2 (4)
Fukuyama	1 (2)
Ullrich disease	1 (2)
Unspecified	4 (7)
GSD type II (Pompe disease)	1 (2)
End-stage myopathy, unspecified	1 (2)
Tracheostomy	48 (84)
Total duration of HMV (month)	31.1 (1−138)
Age at the last outpatient visit or death (month)	41.1 (4.2−245.8)

Data are expressed as median (range).

* MV directly associated with the start of HMV

HMV = home mechanical ventilation; MV = mechanical ventilation; GSD = glycogen storage disease

**Table 2:** Clinical Characteristics of Patients According to Underlying NMD.

Variable	Neuromuscular disease			
	SMA I (n = 29)	SMA II (n = 4)	CM (n = 14)	CMD (n = 8)
Age at the start of HMV (months)	6.6 (2−26)	34.3 (31−48)	7.8 (3−121)	32.2 (2−158)
Time from confirmed diagnosis to the start of MV* (months)	1 (-6−13.5)	20 (6.2−33.4)	-2.3 (-7−80.8)	-0.5 (-13.4−113.3)
MV*initiation prior to diagnosis (n)	8	0	11	5
Planned HMV (n)	5	0	1	1
Successful home NIV (n)	2	2	2	2
Failed NIV (hospital/home) (n)	4/3	1/0	7/2	2/0
Tracheostomy (n)	27	2	11	6
Total duration of HMV (month)	32.7 (4−138)	57.4 (50−116)	20.6 (1−55)	25.6 (2−88)
Deaths (n)	8	0	4	0
Age at the last outpatient visit or death (months)	39.9 (9.5−155.3)	98.8 (83.8−146.8)	40.2 (4.8−170.6)	101.3 (4.2−245.8)

Data are expressed as median (range).

* MV directly associated with the start of HMV

NMD = neuromuscular disease; SMA = spinal muscular atrophy; CM = congenital myopathy; CMD = congenital muscular dystrophy; HMV = home mechanical ventilation; MV = mechanical ventilation; NIV = noninvasive ventilation

### Age at the start of HMV

The most common diagnosis of patients who started HMV before 12 months of age (n = 38, 67%) was SMA type I (n = 24). The age at the start of HMV was lower in patients with SMA type I (range, 2.5−25.6 months; median, 6.6) than in patients with SMA type II (30.7−47.6; 34.3) (*P* < 0.001) or with a diagnoses other than SMA type I (2.4−158.1; 11.4) (*P* = 0.016).

### The time of confirmed diagnosis and pre-HMV respiratory morbidity

Emergent mechanical ventilation, which was directly associated with the start of HMV, was initiated in 25 patients (44%) before the confirmed diagnosis of NMD. This group included 11 (79%) of 14 patients with congenital myopathy and 8 (28%) of 29 patients with SMA type I.

Five indicators of pre-HMV respiratory morbidity were greater In 25 patients who received emergent mechanical ventilation prior to the confirmed diagnosis than in the 32 other patients; these indicators were the number of extubation trials, the number of pre-HMV episodes of hypoxia, the proportion of pre-HMV hospitalizations, the pre-HMV length of stay in the pediatric intensive care unit, and the pre-HMV duration of mechanical ventilation in the unit. The 2 groups did not differ significantly in age at time of diagnosis or at the start of HMV, which are related to the severity of patient’s neurologic symptoms, nor in post-HMV data indicating post-HMV respiratory morbidity or mortality (Table 3).

**Table 3:** Characteristics of Groups According to Time of Confirmed Diagnosis Relative to Initiation of MV*.

Variable	MV* initiation before confirmed diagnosis (n = 25)	MV* initiation after confirmed diagnosis (n = 32)	*P*-value
Male (n)	14 (56%)	17 (53.1%)	NS
Age (month)—clinical diagnosis	2.2 (0−127)	5.2 (0.4−44.7)	NS
Age (month)—confirmed diagnosis	5.8 (2−127)	5.7 (0−45)	NS
Time (month) from clinical diagnosis to the start of MV*	-0.2 (-4.4−7)	4.7 (0.1−113.3)	<0.001
Age (month)—start of HMV	6.6 (2−128)	10.6 (2−158)	NS
Extubation trial (n)	2 (0−9)	1 (0−5)	0.004
Pre-HMV hypoxia (n)	3.5 (1−24)	1.5 (0−7)	0.003
Proportion of pre-HMV hospitalizations^†^	0.99 (0.02−1.00)	0.17 (0.00−0.70)	<0.001
Pre-HMV PICU stay (day)	104.5 (30−227)	20.5 (0−175)	<0.001
Pre-HMV duration of MV in the PICU (day)	92 (24−214)	15.5 (0−175)	<0.001
Post-HMV hypoxia (n)	1 (0−7)	1 (0−9)	NS
Proportion of post-HMV hospitalizations^§^	0.01 (0−0.52)	0.01 (0−0.39)	NS
Post-HMV PICU stay (day)	0 (0−48)	0 (0−30)	NS
24hr/day of HMV (n)	17 (68%)	23 (71.9%)	NS
Total duration of HMV (month)	14.8 (1−107)	45.5 (4−138)	NS
Death (n)	7 (33.3%)	6 (19.4%)	NS
Age (month)—last outpatient visit or death	30.4 (4.2−140.9)	56.3 (9.5−245.8)	NS

Data are expressed as median (range).

* The MV directly associated with the start of HMV

^†^ Total cumulative days of pre-HMV admissions divided by age (days) at the start of HMV

^§^ Total cumulative days of post-HMV admissions divided by total duration (days) of HMV

SMA = spinal muscular atrophy; MV = mechanical ventilation; NMD = neuromuscular disease; CM = congenital myopathy; CMD = congenital muscular dystrophy; HMV = home mechanical ventilation; PICU = pediatric intensive care unit; NIV = noninvasive ventilation; NS = not significant

### The decision to use HMV

HMV was initiated by a planned decision for 7 patients (12%). Pulmonary function tests were performed prior to making the decision to apply HMV in only one patient with congenital myopathy who was 9 years old; the test revealed a severe restrictive pattern. Polysomnography was not carried out prior to performing planned HMV in any case in the entire study population.

### Changes in respiratory morbidity after HMV

The most common cause of pre- and post-HMV hospitalizations was respiratory tract infection that worsened the underlying neuromuscular respiratory distress (n = 35, 61%). The proportion of post-HMV hospitalizations (0.00−0.52; 0.01) was lower than that of pre-HMV hospitalizations (0.00−1.00; 0.34) (*P* = 0.004). The number of episodes of post-HMV hypoxia (0−9; 1) was smaller than that of pre-HMV hypoxia (0−24; 4) (*P* < 0.001). Post-HMV length of stay in the pediatric intensive care unit was shorter (0–48; 0) than that of pre-HMV (0–227; 82.5) (P < 0.001) (Table 4).

**Table 4:** Changes in Respiratory Morbidity after HMV*.

Variable	Pre- HMV*	Post- HMV*	*P*-value
Episode of hypoxia (n)	4 (0−24)	1 (0−9)	<0.001
Proportion of hospitalizations^†^	0.34 (0.00−1.00)	0.01 (0.00−0.52)	0.004
PICU stay (day)	82.5 (0−227)	0 (0−48)	<0.001

Data are expressed as median (range).

* The MV directly associated with the start of HMV

^†^ Total cumulative days of HMV admissions divided by age (days) at the start of HMV

HMV = home mechanical ventilation; PICU = pediatric intensive care unit; NS = not significant

### Home NIV

NIV was applied in 28 patients (49%) initially or after extubation. Failure to maintain NIV occurred in 20 patients—15 in the hospital and 5 at home. These 20 patients experienced a median of 4.5 (0−11) episodes of hypoxia during NIV and finally received tracheostomy, except for 1 patient, who died. The main causes of NIV failure were air leakage and a large amount of airway secretions. Five patients were able to maintain home NIV only temporarily (for less than 3 months); of those, 4 required re-admission and emergent intubation because of recurrent respiratory failure, and 1 died at home from a sudden respiratory accident (Table 5).

**Table 5:** Characteristics of Patients Able to Maintain Home NIV Temporarily (For Less Than 3 Months).

Characteristic	Patient No.				
	53	55	56	58	61
NMD	SMA I	SMA I	CM-FTD	CM-MT	SMA I
Sex	M	F	F	M	M
Age* (years)	1.1	0.6	0.4	1.1	0.5
Weight* (kg) (percentile)^†^	9 (3−10p)	7.6 (10−25p)	6.9 (10−25p)	8.1 (<3p)	5.4 (<3p)
Planned HMV	Y	N	N	N	N
HMV time/day (hours)	24	24	≥12	night	24
Use of MIE	Y	Y	N	Y	Y
Feeding	PEG tube	NG tube	NG tube	PEG tube	NG tube
Hypoxia^§^ (n)	4	4	7^¶^	1	9
Cause of NIV failure	Leakage, secretion, dys-synchrony	Leakage, secretion, dys-synchrony	death by aspiration during feeding	Leakage, secretion	Leakage, secretion, dys-synchrony
Home NIV duration (months)	2.4	0.7	2.2	2.4	1.2

* At start of HMV

^†^ Body weight percentile for age

^§^ Episode of hypoxia that occurred during home NIV

^¶^ Including the episode that caused death

NIV = noninvasive ventilation; NMD = neuromuscular disease; SMA = spinal muscular atrophy; CM = congenital myopathy; FTD = fiber type disproportion; MT = myotubular; HMV = home mechanical ventilation; MIE = mechanical in-exsufflator; PEG = percutaneous endoscopic gastrostomy; NG = nasogastric

All 8 patients who succeeded in tolerating the initial 3 months of home NIV were able to maintain it continuously (Table 6). The group included 50% (2 of 4) of the patients with SMA type II, but only 7% (2 of 29) of the patients with SMA type I. Each of these patients, however, had experienced 2 episodes of hypoxia during less than 6 months of HMV up to their last outpatient visit.

**Table 6:** Characteristics of Patients with Successful NIV.

Characteristic	Patient No							
	12	14	31	34	39	47	59	64
NMD	SMA II	CMD-MN	SMA II	CM	CMD-Ullrich	CM-type I FA	SMA I	SMA I
Sex	F	M	F	F	M	F	F	F
Age* (yr)	2.6	13.2	2.8	10.1	9.4	5	1.2	0.7
Weight* (kg) (percentile)^†^	9.4 (<3p)	U	10.7 (<3p)	34.6 (50−75p)	46.1 (90−97p)	8.2 (<3)	8.1 (<3p)	6.4 (3−10p^§^)
Planned HMV	N	Y	N	N	N	Y	Y	Y
HMV time/day (hr)	≥12	<12	night	night	night	night	24	night
Use of MIE	N	U	Y	N	Y	Y	Y	Y
Hypoxia^¶^ (n)	1	0	0	0	0	0	2	2
Feeding	oral	oral	oral	oral	oral	oral	PEG tube	NG tube
HMV duration (yr)	9.7	7.3	4.2	4.1	3.0	2.0	0.6	0.3

* At the start of HMV

^†^ Body weight percentile for age

^§^ Body weight percentile for the corrected age

^¶^ Episode of hypoxia that occurred during home NIV

NIV = noninvasive ventilation; NMD = neuromuscular disease; SMA = spinal muscular atrophy; CMD = congenital muscular dystrophy; MN = merosin-negative; CM = congenital myopathy; FA = fiber atrophy; HMV = home mechanical ventilation; MIE = mechanical in-exsufflator; U = unknown; PEG = percutaneous endoscopic gastrostomy; NG = nasogastric

The age at the start of HMV was higher in the successful home NIV patient group (7.9−158.1; 46.7 months) than in the other group (2.4−127.7; 6.9 months) (*P* < 0.001), and planned HMV was provided more frequently in the successful home NIV group (4 out of 8) than in the other groups (1 out of 20 patients who failed to adjust to NIV and 2 out of 29 who never tried NIV) (*P* = 0.005).

### Invasive HMV and complications related to tracheostomy

Tracheostomy was performed in 48 patients, of whom 29 received primary invasive HMV without a trial of NIV and 19 failed to maintain NIV. The most common diagnosis of patients who received primary invasive HMV was SMA type I (n = 20; *P* = 0.021).

Complications related to tracheostomy were evaluated by bronchoscopic exams in 28 of the 48 patients. The results revealed no abnormal finding (n = 20), granulation requiring surgical treatment (n = 7), and tracheal wall erosion (n = 1).

### The modes and the dependence time on HMV

The most common mode of ventilation was pressure support mode with bi-level positive airway pressure and back-up mandatory ventilation, which is supposed to be delivered in case of apnea (n = 48). Forty patients (70%) received HMV all day, whereas 11 (19%) received HMV only during sleep. Nocturnal plus intermittent daytime HMV was required in 3 patients (5%) for less than 12 hours a day and in 2 patients (4%) for more than 12 hours a day. None of the patients in the successful home NIV group required all-day HMV except for 1 patient with SMA type I.

### Weaning from HMV

Weaning from HMV, or reduction in the dependency on HMV, was achieved in 5 patients, all of whom received invasive HMV. The only patient who was successfully weaned had congenital myopathy (nemaline rod). She had received HMV at 8 months of age but lived without mechanical ventilator support after 11 months of age until her last outpatient visit at 91.8 months of age. She was excluded from the analysis of post-HMV data because of the extremely short duration of HMV compared with the relatively long follow-up period.

Reduction in HMV dependency was successful in 4 patients. One female patient with SMA type I (without a survival motor neuron gene mutation and with mitochondrial DNA depletion) received HMV for less than 12 hours a day from 6−31 months of age and again during the nighttime at the age of 56.4 months. A male patient with congenital muscular dystrophy received HMV all day from the age of 6 months, and a reduction to nighttime use was successful at 12 months of age. A male patient with congenital myopathy (fiber type disproportion) received HMV all day starting at 10.8 months, and a reduction to nighttime use was successful at 19.2 months of age. A male patient with congenital myopathy (type I atrophy) received HMV all day from the age of 7.2 months, and a reduction to less than 12 hours a day was successful at 24 months of age.

### Outcomes

The overall cumulative survival rate was 60% (n = 39). We excluded 5 patients from the survival analysis who were lost to follow-up. Among the 13 patients who died, 7 died of sudden respiratory failure or aspiration that occurred at home and 3 died of sepsis (Table 7).

**Table 7:** Characteristics of Patients who Died.

Patient No.	NMD	Sex	Age* (yr)	Tracheostomy (^†^)	HMV time/day	Feeding	Hypoxia (n)^§^	Cause of death (^¶^)
3	GSD II	F	6.0−6.2	Y (N)	24	oral	1	RF (home)
4	SMA I	M	2.1−6.7	Y (G)	24	PEG tube	1	RF (U)
8	SMA I	F	0.3−2.1	Y (N)	24	NG tube	2	sepsis (home)
13	CM-MT	M	0.3−3.3	Y (N)	24	NG tube	1	U (U)
17	SMA I	M	0.3−1.7	Y (U)	24	NG tube	1	U (U)
18	SMA I	M	0.6−4.0	Y (U)	24	PEG tube	1	RF (home)
22	CM-FTD	F	0.5−1.9	Y (U)	24	NG tube	1	sepsis (home)
24	CM	M	0.8−5.5	Y (N)	night	oral	3	obstruction^ǂ^ (home)
27	SMA I	M	0.3−4.8	Y (U)	24	PEG tube	1	T-tube obstruction (home)
32	SMA I	F	0.6−1.7	Y (U)	24	NG tube	1	sepsis (home)
33	SMA I	F	0.7−3.3	Y (U)	24	PEG tube	2	sepsis (home)
35	SMA I	F	0.4−1.6	Y (U)	24	NG tube	1	RF (home)
56	CM-FTD	F	0.4−0.7	N	≥12	NG tube	7	aspiration (home)

* Age at start of HMV−age at death

^†^ Tracheostomy-associated complication (N, no abnormal finding; G, granulation; U, not evaluated or unknown)

^§^ Post-HMV episode of hypoxia, including episode that caused death

^¶^ Where event occurred

^ǂ^ Sudden airway obstruction other than tracheal tube obstruction

NMD = neuromuscular disease; HMV = home mechanical ventilation; GSD = glycogen storage disease; RF = sudden respiratory failure; SMA = spinal muscular atrophy; PEG = percutaneous endoscopic gastrostomy; U = unknown; NG = nasogastric; CM = congenital myopathy; MT = myotubular; FTD = fiber type disproportion

Thirteen patients were able to speak, whereas 14 were not. Seven patients who were younger than 14 months at the last outpatient visit and 23 patients whose ability to verbalize was not investigated at the last outpatient visit were excluded from this analysis. The verbalization rate was higher in the successful home NIV group (6 out of 7) than in the invasive HMV group (7 out of 20) (*P* = 0.002).

Fourteen patients were able to eat orally, whereas 20 required a nasogastric tube and 21 a gastrostomy. The rate of oral feeding was higher in the successful home NIV group (6 out of 8) than in the invasive HMV group (8 out of 47) (*P* = 0.002). The body weight percentile for the age of each patient, as measured at the last outpatient visit, was still insufficient (range, <3rd to >97th; median, 14th) and did not increase significantly compared with that measured at the start of HMV (<3rd to 90th-97th; 8th).

## Discussion

Although HMV has played an important life-supporting role in some children with childhood-onset hereditary NMD [2, 5, 11, 13], the decision to use HMV with or without tracheostomy for children, especially infants, is challenging for pediatric pulmonologists, neurologists, and the patients’ families. In particular, the cause and prognosis of respiratory failure are unclear when the failure precedes the diagnosis, and the decision to apply HMV in such cases takes more time.

In this study, 5 indicators of respiratory morbidity or medical costs were greater in the 25 patients who received mechanical ventilation with the start of HMV prior to a confirmed neuromuscular diagnosis, suggesting that an early confirmed diagnosis for infants and young children who are experiencing respiratory failure and exhibiting abnormal neurologic signs such as hypotonia and motor developmental delay can reduce further respiratory morbidity. If these children require repetitive admissions or emergent mechanical ventilation, pediatric neurologists should decide whether to perform genetic studies for a confirmative diagnosis [12]. Until the diagnosis is confirmed, pediatric pulmonologists should optimize mechanical ventilation, and they should attempt to wean the patient from the ventilator in a manner that is careful and not excessive.

That respiratory tract infection was the most common aggravating factor of respiratory distress in this study, resulting in hospital admissions, was consistent with previous reports [18]. Because the proportion of post-HMV hospitalizations was lower than that of pre-HMV hospitalizations, we propose that proper HMV for patients with NMD contributes to a reduction in respiratory morbidity. According to our study, HMV also contributed to prolonging the life of children with NMD. Those with SMA type I (n = 29) survived until 9.5−155.3 (median; 39.9) months of age and only 3 died before 24 months of age, which, without respiratory support, is regarded as the life span limit in such patients [11, 12].

The issue of planned HMV is mainly relevant for SMA type II, which tends to be diagnosed long before respiratory symptoms occur [17, 19]. Serial physical exams, blood gas analyses, chest radiograph, pulmonary function tests, polysomnography, and thorough discussions with the family are critical for the planned decision of HMV [17, 20–22]. Unfortunately, because of the poor compliance of young children, especially those with a moderate or severe degree of respiratory distress, pulmonary function tests were performed at a low rate in our study, and polysomnography was not performed at all (it is not commonly used for disabled children at our center). In general, we paid more attention to symptoms, physical exams, chest radiographs, morning blood gases, and the family’s opinion than to other factors when we decided whether to apply HMV for children with NMD. Nocturnal oxygen saturation monitoring plus morning blood gases are usually performed as simple assessments of sleep-disordered breathing in young children with NMD at our center [17, 23]. Regular pulmonary function tests and polysomnography for cooperative children with NMD are required for our center to establish more objective indications to reduce the rate of emergent unplanned HMV [21, 24].

In this study, planned HMV was provided more frequently to the successful home NIV group than to the other groups. Compared with invasive ventilation, NIV is thought to be more applicable and suitable for the strategy of timely planned HMV because it does not require surgical tracheostomy and can be applied intermittently or on a trial basis, if needed [20]. Planned home NIV is expected to reduce the frequency of emergent invasive HMV by detecting and supporting patients who require respiratory support before it is too late.

This study also showed, however, that young and small patients had difficulty tolerating NIV. All 5 patients who received home NIV temporarily rather than continually were less than 18 months of age, including an infant who died during home NIV. Otherwise, most patients in the successful home NIV group were more than 18 months of age, except for 2 patients with SMA type I whose follow-up duration is not yet sufficient.

The most common causes of failure to maintain NIV were air leakage and a large amount of airway secretions. Air leakage can be a serious problem if a patient depends heavily on HMV. All patients in this study needed bi-level positive airway pressure, which requires a mask with a better fit than simple continuous positive airway pressure. Although several mask sizes are available, it is not easy to find one suitable for infants and young children who have faces of various sizes and shapes [20]. Further, a nasogastric feeding tube aggravates air leakage by creating a gap between the mask and the patient’s face. In addition, infants and young children usually require full-face masks rather than nasal masks, which they cannot tolerate because of air leaks that occur during crying. Thus, they tend to experience discomfort more often than adults, who tolerate relatively inconvenient nasal masks [25]. Long-term use of a full-face mask results in complications such as pressure sores on the face and aerophagia [26]. Fortunately, since the Korean Ministry of Food and Drug Safety started to import small masks in 2012, they are now commercially available and we have attempted to apply home NIV to infants more aggressively than in the past. The success rate of home NIV for infants with NMD between 2012 and 2013, however, was only 29%, and air leakage was the major cause of NIV failure (Tables 3 and 5). Further technological development of an NIV interface for pediatric patients is required.

Problems caused by airway secretions are inevitable, because most patients with NMD are not able to cough effectively, which interrupts airway secretion drainage. Moreover, coexisting bulbar dysfunction increases the risk of aspiration [1]. Intermittent nasopharyngeal suction and physiotherapy using a mechanical in-exsufflator are needed to cope with these difficulties [27]. Although intensive medical training of caregivers might increase the success rate of home NIV, that is not likely to be a decisive factor because NIV could not be maintained in another 15 patients with a severe pulmonary condition in the pediatric intensive care unit.

Invasive HMV through a tracheostomy helps reduce air leakage and allows for easy removal of sputum by tracheal suction. Tracheostomy is also beneficial for patients with upper airway obstruction caused by a congenital anomaly or an acquired injury related to previous endotracheal intubations [28, 29]. Tracheostomy, however, can cause serious complications (e.g., local infection of the stoma, granulation and erosion of the tracheal mucosa, sudden obstruction of the tracheal tube, and an increase in airway secretion caused by tracheal tube placement) [8, 28, 30] and can interfere with language development [13].

According to our investigation, both invasive and noninvasive HMV have merits and demerits, so it is important that the choice consider the condition of the individual patient. We propose that invasive HMV may be more suitable for patients who cannot tolerate NIV because they are too young to obtain well-fitted masks and require high-pressure or long-duration HMV for up to 24 hours a day [10]. Patients who have severe bulbar dysfunction and a large amount of airway secretions or recurrent atelectasis caused by secretions in spite of frequent nasopharyngeal suction and the use of a mechanical in-exsufflator are also appropriate candidates for invasive HMV [10, 31]. Home NIV, however, is a better choice if the patient is expected to be weaned from mechanical ventilation later or depends on ventilator support intermittently [8, 17, 31].

HMV weaning is possible in children with certain types of NMD, such as congenital myopathy, when the underlying neuromuscular condition improves and appropriate growth is achieved by nutrition [32–34]. In this study, 21% (n = 3) of children with congenital myopathy showed an improved respiratory condition. Among them, one was weaned from HMV and showed steady neuromuscular condition development during the 7-year follow-up period. Reducing the dependence time on HMV was successful in 2 others at the ages of 19.2 and 24 months and with body weights of 10.4 and 11.7 kg, respectively. We expect that these patients will be weaned from HMV in the future.

Although accumulated experience and technological developments have improved HMV safety, the procedure is still intrinsically risky because the patient is ventilated out of the hospital and the capacity for respiratory compensation is poor. Sudden respiratory failure was the leading cause (54%) of death in this study. Most of the patients who died from sudden respiratory accidents were found dead at home unexpectedly and the families did not witness the course of deterioration. Sudden respiratory accidents can be caused by tracheostomy-related problems (e.g., extubation or obstruction of the tracheal tube) [35] or aspiration, especially in patients receiving NIV [16], and ventilator-related problems (e.g., equipment or power failure, disconnection) [36]. A home-monitoring system (e.g., pulse oximetry and a home-ventilator alarm system) and caregiver education are important for minimizing the potential risks of HMV, but it is difficult to eliminate the risks completely [36, 37]. To prevent sudden airway problems complicated by tracheostomy, patients with a tracheostomy should be examined regularly through bronchoscopy and provided with surgical treatment if needed [30]. In this study, the rate of regular airway examinations was only 58%, the result of poor compliance or omissions by clinicians. Among the 12 patients with tracheostomy who died, 7 had either never received a bronchoscopic examination, or their examination history was unknown, and of them, 3 died suddenly from airway secretions or unknown causes.

The second most common cause of death was infection. This can be prevented by providing education about clean handling of the tracheal tube and ventilator circuit, clean suction techniques, and adequate hand hygiene at all times [36, 38].

In conclusion, HMV is an effective therapy for children with childhood-onset hereditary NMD because it improves respiratory morbidity and prolongs life spans. Infants and young children with SMA type I can be helped by an early diagnosis and timely application of HMV. Given the merits and demerits of invasive and noninvasive HMV, clinicians should select the best option of respiratory support while considering each patient’s age and tolerance to NIV trial. Systematic follow-up guidelines provided by a multidisciplinary team and a specialized program are needed to enhance the quality of HMV and to provide practical information for the increasing population of home-ventilated children.
